# Enhanced Palatal Wound Healing with Leucocyte- and Platelet-Rich Fibrin After Free Gingival Graft Harvesting: A Prospective Randomized Controlled Clinical Trial

**DOI:** 10.3390/jcm14031029

**Published:** 2025-02-06

**Authors:** Serap Gulsever, Sina Uckan

**Affiliations:** 1 Department of Oral and Maxillofacial Surgery, School of Dentistry, İstanbul Medipol University, Atatürk Bulvarı No:27, Unkapanı, Fatih, 34083 İstanbul, Turkey; 2 Department of Oral and Maxillofacial Surgery, School of Dentistry, İstanbul Medipol University, TEM Avrupa Otoyolu Göztepe Çıkışı No:1, Bağcılar, 34214 İstanbul, Turkey; isuckan@medipol.edu.tr

**Keywords:** complications, free gingival graft, leukocyte- and platelet-rich fibrin, wound healing

## Abstract

**Background/Objectives:** Autogenous palatal free gingival graft (FGG) harvesting presents challenges for patients due to the increased risk of postoperative morbidity related to a second intraoral surgical wound that heals with secondary intention. This parallel-group, randomized, controlled, open-label trial aimed to evaluate the efficacy of the application of leukocyte- and platelet-rich fibrin (L-PRF) membrane to the palatal donor site on wound healing, hemostasis, and pain control after FGG harvesting. **Methods:** Twenty-eight adult patients with insufficient attached gingiva underwent soft tissue augmentation using FGG harvested from the palate at the Department of Oral and Maxillofacial Surgery, Baskent University, Turkey. Patients were randomized to either an L-PRF group or a control group. In the L-PRF group, the L-PRF membrane was sutured to the donor sites, whereas in the control group, donor sites healed by secondary intention. Postoperative evaluations were conducted on days 1, 3, 5, and 7 and at weeks 2, 3, 4, 5, and 6. Donor sites were evaluated clinically for pain, burning sensation, bleeding, wound healing, and color match to adjacent tissues. Donor site wound areas were analyzed using digital images. **Results:** Two patients were excluded from the analysis due to loss of contact, leaving 26 (*n* = 13, *n* = 13) patients for analysis. Donor site pain and burning sensation were significantly lower in the L-PRF group compared to the control group during the first two postoperative weeks (*p* < 0.001). Bleeding was significantly lower in the L-PRF group on postoperative days 1 and 3 (*p* < 0.001). Clinical healing index scores were significantly higher in the L-PRF group at weeks 3 and 4 (*p* < 0.001). Additionally, palatal wound area reductions from baseline were significantly greater in the L-PRF group at all follow-up intervals (*p* < 0.001). **Conclusions:** The application of an L-PRF membrane to palatal donor wounds after FGG harvesting significantly reduces postoperative pain, decreases bleeding, and accelerates healing, providing a valuable autologous biomaterial for enhanced wound healing and improved patient comfort.

## 1. Introduction

Soft tissue grafting techniques using autogenous free gingival grafts (FGG) and connective tissue grafts are reliable and predictable surgical procedures commonly used to increase the dimensions of keratinized gingiva. These techniques are indicated to address shallow vestibular depth, gingival recession defects, inadequate peri-implant attached gingiva, and post-extraction soft tissue deficiencies prior to immediate implant placement or bone augmentation [1].

Free soft tissue grafts can be harvested from various intraoral sites, including the palate, maxillary tuberosity, buccal mucosa, and edentulous ridge. The maxillary tuberosity provides relatively thick tissue and less postoperative pain but has limited availability and proximity to anatomical structures [1]. The edentulous ridge provides readily available keratinized tissue in edentulous patients but is limited by tissue quantity and thickness. Buccal mucosa offers a good color match, but thin keratinized tissue is prone to shrinkage. The choice depends on several factors, including the amount of tissue required, the characteristics of the recipient site, and the patient’s anatomy and preferences, requiring careful consideration to achieve optimal results.

The keratinized gingiva of the hard palate in the premolar/molar region is the most commonly used donor site [2]. However, harvesting tissue from the palate presents challenges for patients, including a second intraoral surgical wound and an increased risk of postoperative morbidity [3]. The palatal wound, which typically heals over two to four weeks by secondary intention, often causes significant patient discomfort due to pain, burning sensation, prolonged bleeding, and delayed healing, often caused by secondary irritation [4].

Clinicians prioritize rapid and uncomplicated wound healing after oral surgical procedures to minimize postoperative morbidity and preserve patients’ quality of life. A variety of biomaterials and therapeutic techniques such as gelatin sponges, collagen sponges, collagen membranes, oxidized cellulose, tissue adhesives, ozone therapy, low-level laser therapy, herbal products, hyaluronic acid, and platelet concentrates have been employed to minimize postoperative complications and promote donor site healing after palatal soft tissue graft harvesting [5,6].

Platelet concentrates produced by centrifugation of autologous blood contain high concentrations of platelets and growth factors [7]. Whitman [8] described the use of platelet-rich biomaterials in oral and maxillofacial surgery in 1997, which has led to the development of platelet concentrates ranging from first-generation products, such as growth factor-rich plasma and platelet-rich plasma (PRP), to second-generation products, such as pure platelet-rich fibrin (PRF), leukocyte- and platelet-rich fibrin (L-PRF), advanced platelet-rich fibrin (A-PRF), injectable platelet-rich fibrin (i-PRF), and titanium-prepared platelet-rich fibrin (T-PRF).

Leukocyte- and platelet-rich fibrin (L-PRF), a second-generation platelet concentrate, was developed for oral and maxillofacial surgery in 2001 by Choukroun et al. Polymerization without any biochemical activation occurs in a manner comparable to the natural coagulation process, resulting in the formation of a strong fibrin matrix that is resistant to rapid resorption [9,10]. Compressing this fibrin matrix creates a strong, suture-resistant membrane that can be fixed to the wound edges and provides a stable barrier against the oral environment. L-PRF contains platelets, a rich array of growth factors (vascular endothelial growth factor (VEGF), insulin-like growth factor-1 (IGF-1), platelet-derived growth factor (PDGF), transforming growth factor-beta (TGF-β), fibroblast growth factor (FGF), and epidermal growth factor (EGF)), leukocytes, cytokines, and circulating stem cells, providing a rich regenerative environment. It is a simple and cost-effective method of trapping growth factors and cytokines in the fibrin network for release over time [9,11]. The sustained release of growth factors from the L-PRF membrane over a four-week period stimulates cell migration, proliferation, and differentiation. In addition, leukocytes act as anti-infective and immunoregulatory agents, and cytokines contribute to angiogenesis, collagen formation, and endothelial cell function [8,10].

While L-PRF has shown promise in several applications in regenerative dentistry, randomized controlled clinical trials evaluating L-PRF membrane as an adjunct for donor site wound healing in FGG procedures are limited in the literature [5,6,12,13]. This study aimed to evaluate the efficacy of L-PRF membrane as a palatal wound dressing in promoting wound healing, hemostasis, and pain control at palatal donor sites following FGG procedures.

The hypothesis of this study was that applying leukocyte- and platelet-rich fibrin membrane to the palatal donor site after free gingival graft harvesting would significantly improve wound healing and enhance patient comfort by reducing postoperative pain and bleeding, in comparison to allowing the donor site to heal with secondary intention.

## 2. Materials and Methods

### 2.1. Study Design and Participants

This prospective, parallel-group, randomized, controlled clinical trial was designed to evaluate the effects of L-PRF membrane used as a palatal wound dressing following FGG harvesting on postoperative pain, burning sensation, bleeding, and wound healing, according to the CONSORT guidelines. The study protocol was approved by the Baskent University Institutional Review Board Ethics Committee (ethical approval no: D-KA13/15, approval date: 3 January 2014). This study was conducted in accordance with the World Medical Association Declaration of Helsinki (2013 revision) and was retrospectively registered at ClinicalTrials.gov (NCT06777069, registration date: 14 January 2025). All participants were recruited from patients seeking care at the Department of Oral and Maxillofacial Surgery, Faculty of Dentistry, Baskent University, Ankara, Turkey, between January and August 2014. Before enrollment, participants received detailed verbal and written information about the purpose, nature, potential risks, and benefits of the study and provided written informed consent.

A priori power analysis was conducted using NCSS-PASS 2000 software (Number Cruncher Statistical Systems, Kaysville, UT, USA) to determine the necessary sample size. A sample size of 13 participants per group was determined to achieve 85% power and a 5% alpha level, assuming a clinically significant difference of at least 0.8 points in VAS pain scores between the control and experimental groups at any follow-up time. The 0.8-point difference threshold was based on the findings of Shanmugam et al. [14].

Twenty-eight adults requiring soft tissue augmentation with an FGG due to inadequate keratinized attached gingiva were included in this study.

The inclusion criteria were as follows:

- Patients at least 18 years of age.
- Clinical indication for gingival augmentation with an FGG to treat shallow vestibular depth or gingival recession.
- Patients with no smoking habit.
- Patients with no known systemic disease.
- Patients with no periodontal disease or other oral health problems. Patients able to provide informed consent and able to attend all follow-up assessments.

The exclusion criteria were as follows:

- Patients with systemic medical conditions that may compromise wound healing (e.g., uncontrolled diabetes mellitus, hypertension, blood disorders, rheumatoid arthritis).
- Patients receiving medications that may affect periodontal tissues (e.g., antibiotics, immunomodulatory drugs, steroids, non-steroidal anti-inflammatory drugs) within the last 3 months before surgery.
- Patients receiving anticoagulant and antiaggregant therapy.
- Smokers.
- Pregnancy or lactation.
- History of palatal FGG harvesting.

**Figure 1:**
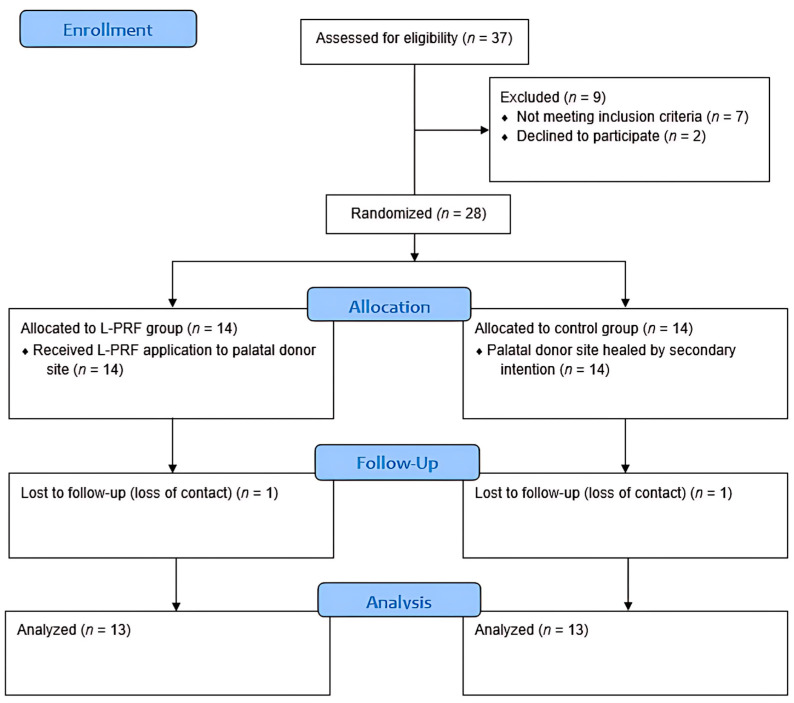
Flow diagram of the study, L-PRF: leukocyte- and platelet-rich fibrin.

Participants were randomly assigned to either an intervention group (*n* = 14), where L-PRF was applied to the donor site, or a control group (*n* = 14), where the donor site healed by secondary intention without L-PRF application. An external researcher performed block randomization using a computer-generated sequence. Allocation concealment was ensured using sealed opaque envelopes. Participant enrollment, randomization, allocation, intervention completion, and analysis were presented in the CONSORT flow diagram (Figure 1).

### 2.2. Surgical Procedures

All participants received professional periodontal prophylaxis and detailed oral hygiene instructions. They were instructed to rinse with 0.12% chlorhexidine gluconate mouthwash (Kloroben, Drogsan, Ankara, Turkey) twice daily for one week prior to surgery. An alginate impression of the maxilla was taken, and an acrylic palatal stent was made to protect the donor area from trauma during the postoperative period.

All procedures were performed by a single clinician (S.U.) to reduce variability in surgical technique. Patients were rinsed with a 0.12% chlorhexidine gluconate solution for 30 s prior to the procedure to reduce oral bacteria and the risk of infection. Articaine 4% with 0.001% epinephrine (Ultracain^®^ D-S Forte) was administered to the palatal donor and recipient sites. Palatal injection was performed by administering 0.3 mL of anesthetic solution over a period of 20 s using a standard dental syringe and a 27-gauge needle. Immediately following palatal anesthesia, patients rated the pain they experienced during the injection using a visual analog scale (VAS) ranging from 0 (no pain) to 10 (the worst pain imaginable). This initial rating was recorded as a VAS1 to assess each patient’s pain threshold.

After achieving adequate anesthesia, a recipient site was prepared to receive the graft in areas lacking sufficient attached gingiva. This procedure involved an incision at the mucogingival junction followed by blunt dissection to separate the epithelium from the underlying connective tissue, creating a vascular bed for graft integration and survival. A sterile paper template was created to match the dimensions of the recipient site, and then the site was covered with moist, sterile gauze.

The paper template was positioned on the palatal mucosa in the premolar/molar region, and the graft margins were outlined with a scalpel (No. 15 blade). Care was taken to avoid critical anatomical structures such as the palatal rugae and greater palatine foramen to minimize potential complications, including bleeding, nerve damage, and postoperative discomfort. Partial-thickness incisions were made perpendicular to the palatal bone, ensuring the blade remained superficial to the periosteum. A 1–2 mm thick graft consisting of epithelium and a thin layer of connective tissue was carefully harvested by moving the blade parallel to the mucosal surface. A moist, sterile gauze was applied to the palatal wound with moderate pressure for one minute to control initial bleeding. Immediately after gauze removal, donor site bleeding was scored as follows: 0 (no bleeding), 1 (bleeding as a leakage), 2 (moderate bleeding), and 3 (severe bleeding). Finally, the baseline donor site photographs were obtained immediately following coagulation.

The FGG was carefully oriented on the recipient site, with the epithelium facing outward and the connective tissue in contact with the recipient bed. It was then sutured using 5-0 bioabsorbable polyglactin sutures to ensure close contact between the graft and the underlying tissue to prevent any movement that could compromise revascularization and lead to graft failure. A moist gauze was applied with gentle pressure for two minutes to promote initial graft adhesion through the formation of a thin fibrin clot.

In the L-PRF group, 10 mL of venous blood was drawn from each patient’s antecubital vein using sterile, anticoagulant-free, glass tubes and immediately centrifuged (PC-02 centrifuge, Process Ltd., Nice, France) at 3000 rpm (RCF: ~400 g, fixed-angle: 33°) for 10 min [10]. After centrifugation, the clot was allowed to stand for about 10 min before being removed from the tube to obtain a more organized fibrin matrix. A fibrin clot was structured in the middle of the tube, just between the red corpuscles at the bottom and the acellular plasma at the top. The L-PRF layer was isolated and compressed into a flexible membrane using its special box. The L-PRF membrane was shaped to fit the donor site wound and placed as a single layer, ensuring complete contact with the underlying tissue. It was secured at the wound corners using 5-0 bioabsorbable polyglactin sutures to minimize displacement during the healing process.

### 2.3. Postoperative Care and Follow-Up

All patients were instructed to wear their palatal stents, avoid hot, hard, acidic, and spicy foods, and avoid brushing the surgical sites for two weeks following surgery. They were also prescribed a regimen consisting of amoxicillin and clavulanic acid (Augmentin BID, GlaxoSmithKline, İstanbul, 1000 mg, twice daily for five days), naproxen sodium (Apranax Forte, Abdi İbrahim, İstanbul, 550 mg, twice daily for five days), and 0.12% chlorhexidine gluconate mouthwash three times daily for one week. Sutures were removed from both the donor and recipient sites one week after surgery.

**Figure 2:**
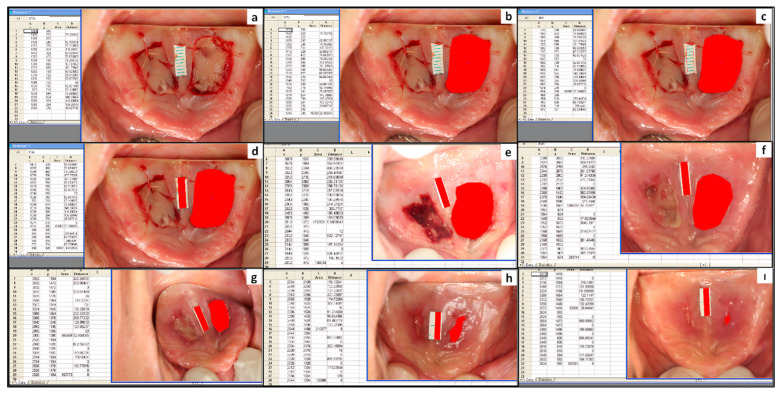
(**a**,**b**) Calculation of the donor site wound area in pixels at baseline. (**c**,**d**) Calculation of the paper ruler area portion in pixels with an actual area of 0.2 cm^2^ at baseline. (**e**–**i**) Calculation of the donor site wound area and ruler area in pixels on days 3, 7, 14, 21, and 28 postoperatively, respectively.

Participants were evaluated on days 1, 3, 5, and 7, and weeks 2, 3, 4, 5, and 6 postoperatively to monitor wound healing and complications at the palatal donor site. An examiner (S.G.) recorded subjective parameters (pain, burning sensation), clinically evaluated objective parameters (postoperative bleeding, wound healing, and color matching with the adjacent healthy tissue), obtained donor site photographs, and performed wound area measurements using digital image analysis. Objective parameters and wound areas were recorded as the average of the scores and areas of the right and left donor site wounds in participants who underwent bilateral palatal graft harvesting to control for inter-individual variability. The evaluations were performed as follows:

- Pain and burning sensation at the donor site were assessed on postoperative day 1 and weeks 1, 2, 3, and 4 using a VAS of 0–10.
- Postoperative bleeding at the palatal donor site was assessed on postoperative days 1, 3, 5, and 7 using a scale of 0 to 3: 0 = no bleeding, 1 = mild bleeding, 2 = moderate bleeding, and 3 = severe bleeding.
- The color matching of the donor site with the adjacent healthy tissue was assessed on postoperative day 1 and weeks 1, 3, and 6 using a scale of 0 to 10: 0 = no match, 10 = perfect match.
- Clinical wound healing at the donor site was assessed at postoperative weeks 1, 2, 3, and 4 and scored from 0 to 5 based on the consistency of the clinical appearance with the expected course of healing. Deviations from the expected appearance were recorded as “−1”. The expected healing course was as follows: Days 1–7: Fibrin covers the wound surface with petechiae at the wound edge; Days 7–14: Decreased fibrin coverage; Days 14–21: Epithelialization of the defect area; Day 21 onwards: Complete healing. Donor site wound areas were measured digitally from standardized photographs taken immediately after palatal graft harvesting, on postoperative day 3, and at weeks 1, 2, 3, and 4. Wound area reductions relative to baseline were calculated in percentages. Photographs were captured using a Canon EOS 60D digital single lens reflex (DSLR) camera with a 100-mm macro lens and a Canon MR-14EX Macro Ring-Lite flash (Canon, Inc., Tokyo, Japan). The lens was positioned at a 45-degree angle to the reflected image of the palate in an occlusal mirror to minimize perspective distortion. During photography, a sterile 1 cm paper ruler with 1 mm markings and a known area of 0.2 cm^2^ was placed adjacent to the wound for calibration. The donor site wound area and ruler area were digitally calculated in pixels using Sigmascan^®^ Pro 5.0 software (SPSS Inc., Chicago, IL, USA). The pixel area of the ruler was calibrated to its actual area in square centimeters, and the actual wound area was calculated using this ratio (Figure 2).

### 2.4. Statistical Analysis

Statistical analysis was performed using SPSS for Windows version 11.5. The Shapiro–Wilk test was used to assess the normality of continuous numerical variables. Descriptive statistics were presented as mean, standard deviation, median, minimum, and maximum for discrete numerical (age), continuous numerical (wound surface area and percentage reduction in wound surface area), and ordinal (pain score, burning sensation score, postoperative bleeding score, color matching score, and clinical healing index score) variables. For nominal variables (gender, indication, recipient site, and donor site), data were presented as frequencies and percentages. The significance of differences between the control and L-PRF groups was assessed using the Student’s *t*-test for discrete numerical variables and the Mann–Whitney U test for continuous numerical and ordinal variables. Nominal variables were analyzed using Pearson’s chi-square, Fisher’s exact, or likelihood ratio tests, as appropriate. Within-group differences between follow-up times for continuous numerical or ordinal variables were assessed using the Wilcoxon signed-rank test. Spearman’s correlation test examined relationships between continuous numerical and ordinal variables. The results were considered statistically significant when *p* < 0.05 unless otherwise stated. However, the Bonferroni correction was applied to control for type I errors in multiple comparisons. The Bonferroni correction compensates for that increase by testing each individual hypothesis at a significant level of *α/m*, where *α* is the desired overall alpha level and *m* is the number of hypotheses or pairwise comparisons.

## 3. Results

A total of 28 patients were treated without any adverse effects. Of these, one patient from each group was lost to follow-up, and data for these patients were not included in this study. Table 1 presents the demographic and clinical characteristics of the 26 participants (13 in the L-PRF group and 13 in the control group) who completed the 6-week follow-up. The mean age of the patients in the L-PRF group (57.1 ± 8.3 years) was statistically significantly lower than that of the control group (66.3 ± 10.7 years) (*p* = 0.021). However, there were no statistically significant differences between the groups regarding gender (*p* = 0.695), indications (*p* = 0.480), recipient sites (*p* > 0.999), and donor site sides (*p* = 0.420).

**Figure 3:**
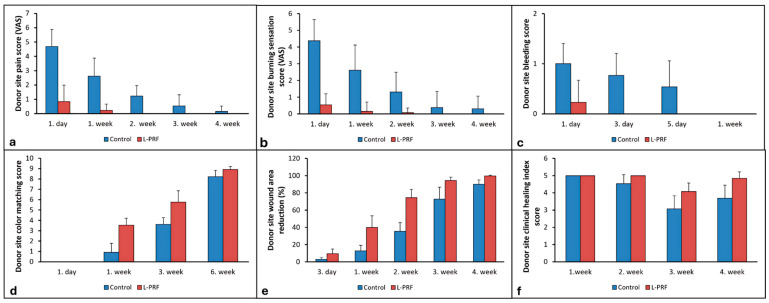
(**a**) Donor site pain scores at follow-up intervals. (**b**) Donor site burning sensation scores at follow-up intervals. (**c**) Donor site bleeding scores at follow-up intervals. (**d**) Donor site color matching scores at follow-up intervals. (**e**) Donor site wound area percentage reductions relative to baseline at follow-up intervals. (**f**) Clinical healing index scores of donor site wounds at follow-up intervals.

Donor site pain scores at follow-up intervals are presented in Table 2 and Figure 3a. The mean VAS1 scores were 2.00 ± 1.63 for the L-PRF group and 2.92 ± 1.94 for the control group, with no statistically significant difference between the groups (*p* = 0.223). Pain scores were significantly lower in the L-PRF group compared to the control group on day 1 and at weeks 1 and 2 postoperatively (*p* < 0.001). In the control group, pain scores decreased over time, with statistically significant differences observed between all follow-up intervals except between weeks 2 and 3 and weeks 3 and 4 (*p* < 0.0025). In the L-PRF group, the pain was resolved by week 2, with no statistically significant differences in the pain scores observed between any two follow-up intervals (*p* > 0.0025).

Donor site burning sensation scores at the follow-up intervals are presented in Table 3 and Figure 3b. Burning sensation scores were significantly lower in the L-PRF group compared to the control group on day 1 and at weeks 1 and 2 postoperatively (*p* < 0.001). In the control group, burning sensation scores decreased over time, with statistically significant differences observed between day 1 and weeks 1, 2, 3, and 4 (*p* < 0.0025). In the L-PRF group, the burning sensation was resolved by week 3, with no statistically significant differences in the burning sensation scores observed between any two follow-up intervals (*p* > 0.0025).

The mean intraoperative bleeding scores were 2.08 ± 0.49 for the L-PRF group and 1.92 ± 0.49 for the control group, with no statistically significant difference between the groups (*p* = 0.579). Donor site bleeding scores at the follow-up intervals are presented in Table 4 and Figure 3c. Bleeding scores were significantly lower in the L-PRF group compared to the control group on days 1 and 3 postoperatively (*p* < 0.001). In the control group, bleeding scores decreased over time, with statistically significant differences observed between day 1 and week 1, and between day 3 and week 1 (*p* < 0.0042), with no bleeding on day 7. In the L-PRF group, the bleeding disappeared by day 3, with no statistically significant differences in the bleeding scores between any two follow-up intervals (*p* > 0.0042).

Color matching scores of the donor site with the adjacent healthy tissue at the follow-up intervals are presented in Table 5 and Figure 3d. The color matching scores were statistically significantly higher in the L-PRF group compared to the control group at weeks 1, 3, and 6 postoperatively (*p* < 0.001, *p* < 0.001, *p* = 0.006, respectively). In the control group, the color matching scores increased significantly between all follow-up intervals except between day 1 and week 1 (*p* < 0.001). In the L-PRF group, scores increased significantly between any two follow-up intervals (*p* < 0.001).

The mean baseline donor site wound area (graft area) was 2.98 ± 0.59 cm^2^ for the L-PRF group and 2.48 ± 1.04 cm^2^ for the control group, with no statistically significant difference between the groups (*p* < 0.0083). Donor site wound areas at the follow-up intervals are presented in Table 6. The wound area decreased significantly between all follow-up intervals except between baseline and day 3 in the control group and between week 3 and week 4 in the L-PRF group (*p* < 0.0017). Percentage reductions in donor site wound area, relative to baseline, are presented in Table 7 and Figure 3e. Intergroup comparisons revealed significantly greater percentage reductions in the L-PRF group compared to the control group on day 3 and at weeks 1, 2, 3, and 4 postoperatively (*p* < 0.0033).

Clinical healing index (CHI) scores of the donor site wound at the follow-up intervals are presented in Table 8 and Figure 3f. CHI scores were significantly higher in the L-PRF group compared to the control group at weeks 3 and 4 postoperatively (*p* = 0.002 and *p* < 0.001, respectively). Intragroup comparisons revealed that the lowest mean CHI scores were observed at week 3 in both groups. In the control group, CHI scores decreased significantly from week 1 to weeks 3 and 4, as well as from week 2 to week 3 (*p* < 0.0042). In the L-PRF group, CHI scores decreased significantly from weeks 1 and 2 to week 3; however, a significant increase was observed from week 3 to week 4 (*p* < 0.0042).

## 4. Discussion

Free gingival grafting, the most common mucogingival procedure for augmenting keratinized gingiva, was introduced by Sullivan and Atkins in 1968 [15]. This technique is considered the gold standard for simplicity, predictability, and satisfactory graft volumes. The palate, particularly the area between the first molar and the canine, is the preferred site for FGG harvesting. However, the FGG technique is associated with high complication rates, particularly at the palatal donor site, which heals by secondary intention, and at the recipient site, where graft survival and avoidance of necrosis are critical. Donor site complications include increased risk of pain, bleeding, infection, tissue necrosis, and reduced quality of life. In FGG procedures, minimizing postoperative complications and patient discomfort is crucial.

The pursuit of improved postoperative clinical outcomes and patient quality of life has led to extensive research into modulating cellular behavior. There has been growing interest in applying biologically active materials to surgical sites to promote wound healing. L-PRF, a second-generation platelet concentrate, has emerged as a promising biomaterial in this context. This autologous biomaterial, obtained by a single-stage centrifugation process without the addition of anticoagulants or synthetic substances, concentrates platelets and cytokines within a fibrin network that acts as a scaffold to promote tissue regeneration [7,16]. Although the efficacy of L-PRF in wound healing has been demonstrated in various applications, controlled clinical studies investigating its effects on oral mucosal wound healing remain limited [5,12]. The palatal donor site heals by secondary intention following FGG harvesting. This characteristic, combined with its superficial and measurable nature, makes it an ideal model for evaluating the effects of biomaterials on intraoral wound healing. This controlled clinical study evaluated the effects of L-PRF on postoperative pain, burning sensation, bleeding, and wound healing at the palatal donor site for six weeks following the FGG procedure.

L-PRF controls bleeding and provides a temporary matrix that protects the blood clot at the injury site. Blood vessels support cellular metabolism by delivering oxygen and nutrients, while activated platelets release essential growth factors contributing to granulation tissue formation. It has been reported that the platelet concentration of L-PRF is seven times that of blood, and it contains cytokines that protect growth factors from proteolysis, extending their release for up to 14 days [17]. In vitro studies have demonstrated that the sustained release of autologous growth factors from L-PRF leads to more pronounced and longer-lasting effects on cellular proliferation and differentiation. Dohan et al. reported that L-PRF provided significant and continuous stimulation of gingival fibroblasts, dermal keratinocytes, preadipocytes, and maxillofacial osteoblasts for 21 days [9]. L-PRF is a suitable biomaterial for superficial skin and mucosal wounds due to its ability to promote rapid angiogenesis and early remodeling of a more resistant connective tissue [18].

Ensuring the comparability of demographic characteristics between patient groups is essential for reliable data analysis in clinical trials. In this study, the distributions of gender, indication, recipient site, and donor site were similar between the control and L-PRF groups. The lack of a significant difference in VAS1 scores recorded during palatal anesthesia further strengthens the reliability of pain score comparisons between the groups.

The subjective nature of pain presents challenges for objective assessment. VAS is a widely accepted method for evaluating pain intensity due to its regular distribution, sensitivity to treatment effects, reproducibility, and applicability [19,20]. The VAS remains a valuable tool despite limitations such as potential inaccuracies due to random marking, timing variations, the patient’s physiological and psychological state, and the influence of previous scores on the same scale. In this study, postoperative pain and burning sensation were assessed using VAS for four weeks, as postoperative discomfort at the donor site typically resolves when the mature keratinized epithelium covers the wound. The analysis of pain and burning sensation VAS scores revealed similar trends. Pain and burning scores gradually decreased over time in both groups. Scores in the L-PRF group were significantly lower than in the control group during the first two weeks, consistent with previous studies [21,22,23]. Hassan et al. [21] reported significantly lower VAS pain scores compared to a hyaluronic acid group at 3 and 7 days and compared to a gelatin sponge control group from 3 to 30 days postoperatively. Similarly, Ozcan et al. [22] observed lower VAS scores between days 1 and 14 compared to a wet gauze compression control. Bahammam [23] also reported lower scores between days 3 and 7 compared to an untreated control. L-PRF application resulted in significantly lower initial postoperative pain scores compared to the control group, as observed in previous studies [22,23,24,25,26]. Due to these lower initial scores, the differences in scores between follow-up intervals in the L-PRF group were not statistically significant. In contrast, the control group had higher initial scores, which decreased with statistically significant differences within the first two weeks of follow-up. L-PRF forms a durable membrane that aids healing when used as a wound dressing, and its rich composition contributes to its therapeutic biomaterial properties. However, whether the observed pain reduction is directly caused by accelerated wound healing or specific components within L-PRF remains unclear.

Complications of soft tissue grafting procedures that result in an open palatal wound are often associated with bleeding during the first postoperative week. Individual patient anatomy, especially the location of the greater palatine foramen, influences the risk of bleeding, necessitating careful consideration when selecting the donor site. This study assessed postoperative first-week bleeding on a scale of 0–3. The lack of significant difference in intraoperative bleeding scores suggests standardized patient bleeding tendencies, allowing a reliable comparison of postoperative bleeding.

Postoperative bleeding typically increases 3–7 days postoperatively due to the formation of new, fragile vessels that make the site susceptible to trauma. Research has reported that delayed bleeding is caused by postoperative irritation or trauma rather than the surgery itself. This study recorded the highest bleeding scores in the first few days of the postoperative period. L-PRF application resulted in significantly lower bleeding scores on days 1 and 3 compared to the control group. Mean bleeding scores on day 1 were 0.23 ± 0.44 for the L-PRF group and 1.00 ± 0.41 for the control group. On day 3, the L-PRF group showed no bleeding, while the control group had a mean score of 0.77 ± 0.44. L-TZF effectively reduced bleeding on day one and eliminated it by day three. The control group showed a gradual decrease in bleeding over five days, with bleeding disappearing by the end of the first week. Our findings demonstrate the significant beneficial effect of L-PRF on early hemostasis, consistent with existing studies [22,27,28] demonstrating the effectiveness of different PRF types in promoting rapid hemostasis in the palatal donor site compared to various control interventions. Ozcan et al. [22] reported significantly less bleeding in the PRF group compared to a wet gauze control, with 0% of the PRF group experiencing bleeding on days 1 and 5, compared to 90.2% and 22% in the control group, respectively. Kızıltoprak and Uslu [27] also reported significantly less bleeding on day 3 in the i-PRF group (16.7% of patients experienced bleeding) compared to a sterile tamponade control group (75% of patients experienced bleeding). Ustaoğlu et al. [28] reported similar results, with %87.5 of the T-PRF group experiencing bleeding on day 1 compared to %100 in the control group, and this difference further increased on day 2, with %12.5 for the T-PRF group and %66.7 for the wet gauze control group. The ability of L-PRF to minimize postoperative bleeding likely results from the synergistic effect of the stable fibrin structure supporting the blood and the sustained release of various growth factors and matrix proteins [29]. Among these, thrombospondin-1 (TSP-1), a glycoprotein, has been reported to play a key role in coagulation pathways by contributing to clot formation and maintaining the integrity of platelet aggregates [9,30,31]. Furthermore, the observation of bleeding mainly during the initial postoperative days in both groups could be linked to the use of palatal plates postoperatively to protect the wound sites from external trauma that could interfere with the healing process or cause increased pain or bleeding. These plates may act as a barrier limiting postoperative bleeding to surgically induced bleeding, which typically occurs within the first three days due to impaired vascular integrity.

Oral wounds generally heal faster and with less scarring than skin wounds due to several factors, such as gingival fibroblasts having superior growth and migration compared to dermal fibroblasts, a greater capacity for organic matrix formation, production of matrix metalloproteinases, and expression of lower levels of pro-inflammatory cytokines. Saliva also contributes to oral wound healing by providing beneficial mediators and maintaining a moist environment [32]. However, the oral cavity presents challenges to surgical wound healing due to plaque accumulation, trauma from mastication, complex oral flora, and difficulties in maintaining postoperative oral hygiene [33]. These potential inhibitory factors underscore the importance of protecting the wound site with a biomaterial to minimize exposure to these adverse effects and promote faster healing.

Wound healing at the palatal donor site has been assessed by various methods, including visual comparison of wound color and texture with the contralateral counterpart, assessment of epithelialization using the hydrogen peroxide bubble test, quantification of fibrin coverage or necrosis within the palatal wound, and qualitative and/or quantitative analysis of clinical color photographs [6]. In the present study, wound healing was assessed using both clinical and photometric methods. The L-TZF group showed a healing appearance significantly closer to the expected appearance at weeks 3 and 4 and had a significantly higher color match at weeks 1, 3, and 6 than the control group. Similarly, Bahammam [24] reported that the untreated control group showed poorer color matching than the PRF group at every time point during the two-month follow-up. Ustaoğlu et al. [29] reported that the T-PRF group showed a better color match than the control group based on VAS scores on days 7 and 14. Hassan et al. [22] reported that the PRF group had the highest healing index scores at all intervals compared to the hyaluronic acid and control groups, with significant differences at 7, 14, and 21 days. The authors also mentioned that the PRF group had a mean index of 4.4 at 30 days, indicating complete palatal wound healing.

Precise wound area measurement is critical, particularly when tracking small changes in the donor area during specific healing periods. A computerized image analysis program enables accurate measurements and reliable comparisons between groups. This method relies on the principle that objects within the same plane of a photograph maintain their proportional size, even if distorted during the photography process. Using a paper ruler with known dimensions facilitated standardized measurements and enabled objective comparisons between the groups in this study. The absence of a statistically significant difference in graft area between the groups confirms that any observed changes in wound area can be attributed to L-PRF rather than variations in initial graft size.

In the present study, both groups exhibited a gradual reduction in wound area over the four weeks. However, the L-PRF group showed more significant reductions from baseline than the control group at all follow-up assessments. At week three, the L-PRF group showed a 94.3 ± 3.9% reduction in wound area compared to a 72.8 ± 13.8% reduction in the control group, suggesting an accelerated healing rate associated with L-PRF treatment. These findings align with existing studies that assessed wound area reductions using digital photographs [22,23,26,27]. Sousa et al. [27] reported that the A-PRF group showed higher palatal wound reduction rates than the gelatin sponge control group at 7, 14, and 30 days. The most significant difference between the two groups was observed at 30 days, with a reduction of 91.5% in the A-PRF group versus 59.0% in the control group. Ozcan et al. [23] found that the PRF group had significantly higher rates of epithelialization at weeks 2 and 3 compared to the wet gauze control group, achieving complete epithelialization by week 3. Hassan et al. [22] noted significant differences among the PRF, HA, and gelatin sponge control groups at 7 and 14 days. However, at 21 and 30 days, statistically significant differences were only observed between the PRF and the control groups, with the PRF group showing the smallest wound area at 30 days. Although not statistically significant, Patarapongsanti et al. [26] reported higher rates of wound area reduction in the PRF group at weeks 2 and 3, with complete epithelialization occurring by week 3.

The results of this study suggest that L-PRF contributes to wound healing by actively promoting tissue regeneration, as evidenced by significantly higher clinical healing index scores and more significant wound area reductions. Additionally, L-PRF effectively manages postoperative complications by significantly reducing pain, burning sensation, and bleeding, thus improving patient comfort and recovery time, addressing a critical need to enhance the overall patient experience. These multiple benefits extend beyond symptom management to active tissue regeneration, in line with the overall goals of regenerative dentistry to restore both form and function. This is particularly crucial in free gingival grafting, where donor site morbidity is a significant concern. This study emphasizes the potential of L-PRF as a valuable tool for enhancing the healing of the palatal donor site following free gingival graft harvesting, offering a minimally invasive and effective approach to improving patient care and overall outcomes.

This study provides compelling evidence for the benefits of L-PRF in palatal wound healing. However, it has limitations, including the relatively small sample size, the inability to blind patients to the use of L-PRF due to ethical concerns regarding blood collecting in the control group, the inability to blind the investigator during digital image analysis and clinical assessment due to the nature of the surgical procedures, the lack of histological analysis, and the absence of baseline platelet count and morphology assessments. The small sample size in this study limits the generalizability of the findings. While the baseline characteristics, including gender, indications, recipient/donor sites, VAS1, and intraoperative bleeding scores, were comparable between groups, the limited number of participants makes it difficult to confidently extrapolate the observed benefits of L-PRF to a larger and more diverse population. The smaller the sample, the higher the risk that the observed effects are due to the specific characteristics of the included participants. Larger, more representative samples in future studies would increase the statistical power and allow for conclusions about the effectiveness of L-PRF in a broader range of patients. Histological analysis was also precluded due to ethical concerns, suggesting future histomorphometric evaluation for a more comprehensive understanding of tissue healing. Platelet counts and morphological assessments were not performed prior to L-PRF preparation because patients with a history of bleeding disorders and use of medications that affect platelet function were excluded from this study, but as this information is based on patient self-report, incorporating these hematological assessments in future studies may improve patient selection and procedure outcomes. Finally, studies exploring the potential synergistic effects of L-PRF with other regenerative modalities, such as growth factors or stem cells, and optimizing L-PRF preparation protocols, may further enhance its clinical efficacy.

## 5. Conclusions

L-PRF significantly improved postoperative outcomes following the harvesting of free gingival grafts. It effectively reduced pain and burning sensation while minimizing postoperative bleeding at the donor site, particularly during the critical early stages of healing. The application of this biomaterial enhanced the wound healing process, as evidenced by a notable reduction in wound area and improved clinical healing indices. The results of this study demonstrated the efficacy of L-PRF as a palatal surgical dressing in promoting wound healing and improving postoperative patient comfort.

## Figures and Tables

**Table 1:** Demographic and clinical characteristics of study participants, including age, gender, indication of free gingival graft procedure, recipient site, and donor site.

	Control (*n* = 13)	L-PRF (*n* = 13)	*p*-Value
Age (years) * (mean ± SD)	66.3 ± 10.7	57.1 ± 8.3	0.021 ^†^
(min-max)	44–76	37–66	
Gender **			0.695 ^‡^
Female	7 (53.8)	6 (46.2)	
Male	6 (46.2)	7 (53.8)	
Indication **			0.480 ^¶^
Shallow vestibular depth	11 (84.6)	13 (100.0)	
Gingival recession	2 (15.4)	0 (0.0)	
Recipient site **			>0.999 ^¶^
Anterior mandible	12 (92.3)	12 (92.3)	
Anterior maxilla	1 (7.7)	1 (7.7)	
Palatal donor site **			0.420 ^‡^
Unilateral	6 (46.2)	4 (30.8)	
Bilateral	7 (53.8)	9 (69.2)	

SD: standard deviation; min: minimum; max: maximum; L-PRF: leukocyte- and platelet-rich fibrin; Data are shown as * mean ± SD or ** number of cases and percentages (%), where appropriate. ^†^ Student’s *t* test; ^‡^ Pearson’s chi-square test; ^¶^ Fisher’s exact test.

**Table 2:** Intragroup and intergroup comparisons of donor site pain scores at follow-up intervals.

	Mean	SD	Median	Minimum	Maximum	*p*-Value ^†^
1. day						<0.001
Control	4.69	1.18	5.0	3.0	6.0	
L-PRF	0.85	1.14	0.0	0.0	3.0	
1. week						<0.001
Control	2.62 ^a^	1.26	2.0	0.0	5.0	
L-PRF	0.23	0.44	0.0	0.0	1.0	
2. week						<0.001
Control	1.23 ^a,b^	0.73	1.0	0.0	3.0	
L-PRF	0.00	0.00	0.0	0.0	0.0	
3. week						0.101
Control	0.54 ^a,b^	0.78	0.0	0.0	2.0	
L-PRF	0.00	0.00	0.0	0.0	0.0	
4. week						0.511
Control	0.15 ^a,b,c^	0.38	0.0	0.0	1.0	
L-PRF	0.00	0.00	0.0	0.0	0.0	

SD: standard deviation; L-PRF: leukocyte- and platelet-rich fibrin; ^†^ For between-group comparisons, the Mann–Whitney U test was employed. According to the Bonferroni correction, results with *p* < 0.010 (i.e., α/m = 0.050/5) were considered statistically significant. On the other hand, the Wilcoxon signed-rank test was used for intra-group analyses. According to the Bonferroni correction, results with *p* < 0.0025 (i.e., α/m = 0.050/20) were considered statistically significant. ^a^: Within the control group, the differences between day 1 and the follow-ups were statistically significant (*p* < 0.0025), ^b^: Within the control group, the differences between week 1 and the follow-ups were statistically significant (*p* < 0.0025), ^c^: Within the control group, the differences between week 2 and the follow-ups were statistically significant (*p* < 0.0025).

**Table 3:** Intragroup and intergroup comparisons of donor site burning sensation scores at follow-up intervals.

	Mean	SD	Median	Minimum	Maximum	*p*-Value ^†^
1. day						<0.001
Control	4.38	1.26	4.0	3.0	6.0	
L-PRF	0.54	0.66	0.0	0.0	2.0	
1. week						<0.001
Control	2.62 ^a^	1.50	3.0	0.0	5.0	
L-PRF	0.15	0.55	0.0	0.0	2.0	
2. week						<0.001
Control	1.31 ^a^	1.18	1.0	0.0	4.0	
L-PRF	0.08	0.28	0.0	0.0	1.0	
3. week						0.511
Control	0.38 ^a^	0.96	0.0	0.0	3.0	
L-PRF	0.00	0.00	0.0	0.0	0.0	
4. week						0.511
Control	0.31 ^a^	0.75	0.0	0.0	2.0	
L-PRF	0.00	0.00	0.0	0.0	0.0	

SD: standard deviation; L-PRF: leukocyte- and platelet-rich fibrin; ^†^ For between-group comparisons, the Mann–Whitney U test was employed. According to the Bonferroni correction, results with *p* < 0.010 (i.e., α/m = 0.050/5) were considered statistically significant. On the other hand, the Wilcoxon signed-rank test was used for intra-group analyses. According to the Bonferroni correction, results with *p* < 0.0025 (i.e., α/m = 0.050/20) were considered statistically significant. ^a^: Within the control group, the differences between day 1 and the follow-ups were statistically significant (*p* < 0.0025).

**Table 4:** Intragroup and intergroup comparisons of donor site bleeding scores at follow-up intervals.

	Mean	SD	Median	Minimum	Maximum	*p*-Value ^†^
1. day						<0.001
Control	1.00	0.41	1.0	0.0	2.0	
L-PRF	0.23	0.44	0.0	0.0	1.0	
3. day						<0.001
Control	0.77	0.44	1.0	0.0	1.0	
L-PRF	0.00	0.00	0.0	0.0	0.0	
5. day						0.019
Control	0.54	0.52	1.0	0.0	1.0	
L-PRF	0.00	0.00	0.0	0.0	0.0	
1. week						>0.999
Control	0.00 ^a,b^	0.00	0.0	0.0	0.0	
L-PRF	0.00	0.00	0.0	0.0	0.0	

SD: standard deviation; L-PRF: leukocyte- and platelet-rich fibrin; ^†^ For between-group comparisons, the Mann–Whitney U test was employed. According to the Bonferroni correction, results with *p* < 0.0125 (i.e., α/m = 0.050/4) were considered statistically significant. On the other hand, the Wilcoxon signed-rank test was used for intra-group analyses. According to the Bonferroni correction, results with *p* < 0.0042 (i.e., α/m = 0.050/12) were considered statistically significant. ^a^: Within the control group, the differences between day 1 and the follow-ups were statistically significant (*p* < 0.001), ^b^: Within the control group, the differences between day 3 and the follow-ups were statistically significant (*p* = 0.002).

**Table 5:** Intragroup and intergroup comparisons of donor site color matching scores at follow-up intervals.

	Mean	SD	Median	Minimum	Maximum	*p*-Value ^†^
1. day						>0.999
Control	0.00	0.00	0.0	0.0	0.0	
L-PRF	0.00	0.00	0.0	0.0	0.0	
1. week						<0.001
Control	0.92	0.86	1.0	0.0	2.0	
L-PRF	3.54 ^a^	0.66	3.0	3.0	5.0	
3. week						<0.001
Control	3.62 ^a,b^	0.65	4.0	3.0	5.0	
L-PRF	5.77 ^a,b^	1.09	6.0	4.0	8.0	
6. week						0.006
Control	8.23 ^a,b,c^	0.60	8.0	7.0	9.0	
L-PRF	8.92 ^a,b,c^	0.28	9.0	8.0	9.0	

SD: standard deviation; L-PRF: leukocyte- and platelet-rich fibrin; ^†^ For between-group comparisons, the Mann–Whitney U test was employed. According to the Bonferroni correction, results with *p* < 0.0125 (i.e., α/m = 0.050/4) were considered statistically significant. On the other hand, the Wilcoxon signed-rank test was used for intra-group analyses. According to the Bonferroni correction, results with *p* < 0.0042 (i.e., α/m = 0.050/12) were considered statistically significant. ^a^: Within the groups, the differences between day 1 and the follow-ups were statistically significant (*p* < 0.001), ^b^: Within the groups, the differences between week 1 and the follow-ups were statistically significant (<0.001), ^c^: Within the groups, the differences between week 3 and the follow-ups were statistically significant (<0.001).

**Table 6:** Intragroup comparisons of donor site wound areas at follow-up intervals.

	Mean	SD	Median	Minimum	Maximum
Baseline					
Control	2.48	1.04	2.3	1.3	4.9
L-PRF	2.98	0.59	3.0	2.2	4.1
3. day					
Control	2.42	1.05	2.2	1.2	4.9
L-PRF	2.71 ^a^	0.60	2.6	1.9	3.8
1. week					
Control	2.14 ^a,b^	0.86	1.9	1.2	4.0
L-PRF	1.79 ^a,b^	0.53	1.8	0.8	2.7
2. week					
Control	1.58 ^a,b,c^	0.62	1.3	0.8	2.9
L-PRF	0.76 ^a,b,c^	0.28	0.8	0.1	1.1
3. week					
Control	0.67 ^a,b,c,d^	0.55	0.6	0.2	2.4
L-PRF	0.17 ^a,b,c,d^	0.10	0.2	0.0	0.4
4. week					
Control	0.24 ^a,b,c,d,e^	0.11	0.2	0.0	0.4
L-PRF	0.01 ^a,b,c,d^	0.02	0.0	0.0	0.1

SD: standard deviation; L-PRF: leukocyte- and platelet-rich fibrin; For within-group comparisons, the Wilcoxon signed-rank test was employed. According to the Bonferroni correction, results with *p* < 0.0017 (i.e., α/m = 0.050/30) were considered statistically significant. ^a^: Within the groups, the differences between the baseline and the follow-ups were statistically significant (*p* < 0.0017), ^b^: Within the groups, the differences between day 3 and the follow-ups were statistically significant (*p* < 0.0017), ^c^: Within the groups, the differences between week 1 and the follow-ups were statistically significant (*p* < 0.0017), ^d^: Within the groups, the differences between week 2 and the follow-ups were statistically significant (*p* < 0.0017), ^e^: Within the groups, the differences between week 3 and the follow-ups were statistically significant (*p* < 0.0017).

**Table 7:** Intergroup comparisons of donor site wound area percentage reductions relative to baseline.

	Mean	SD	Median	Minimum	Maximum	*p*-Value ^†^
3. day-Baseline						0.002
Control	−3.02	1.91	−2.7	−6.6	0.2	
L-PRF	−9.61	5.33	−9.6	−17.2	−1.1	
1. week-Baseline						<0.001
Control	−12.74	6.50	−14.2	−24.0	−4.1	
L-PRF	−40.02	13.47	−43.4	−66.7	−18.8	
2. week-Baseline						<0.001
Control	−35.53	10.07	−33.9	−56.6	−16.0	
L-PRF	−74.47	9.67	−74.2	−97.8	−57.4	
3. week-Baseline						<0.001
Control	−72.77	13.79	−77.2	−91.0	−44.1	
L-PRF	−94.34	3.93	−95.1	−100.0	−83.5	
4. week-Baseline						<0.001
Control	−90.11	4.98	−89.6	−100.0	−81.8	
L-PRF	−99.80	0.64	−100.0	−100.0	−97.7	

SD: standard deviation; L-PRF: leukocyte- and platelet-rich fibrin; ^†^ For between-group comparisons, the Mann–Whitney U test was employed. According to the Bonferroni correction, results with *p* < 0.0033 (i.e., α/m = 0.050/15) were considered statistically significant.

**Table 8:** Intragroup and intergroup comparisons of clinical healing index scores of donor site wounds at follow-up intervals.

	Mean	SD	Median	Minimum	Maximum	*p*-Value ^†^
1. week						>0.999
Control	5.00	0.00	5.0	5.0	5.0	
L-PRF	5.00	0.00	5.0	5.0	5.0	
2. week						0.044
Control	4.54	0.52	5.0	4.0	5.0	
L-PRF	5.00	0.00	5.0	5.0	5.0	
3. week						0.002
Control	3.08 ^a,b^	0.76	3.0	2.0	4.0	
L-PRF	4.08 ^a,b^	0.49	4.0	3.0	5.0	
4. week						<0.001
Control	3.69 ^a^	0.75	4.0	2.0	5.0	
L-PRF	4.85 ^c^	0.38	5.0	4.0	5.0	

SD: standard deviation; L-PRF: leukocyte- and platelet-rich fibrin; ^†^ For between-group comparisons, the Mann–Whitney U test was employed. According to the Bonferroni correction, results with *p* < 0.0125 (i.e., α/m = 0.050/4) were considered statistically significant. On the other hand, the Wilcoxon signed-rank test was used for intra-group analyses. According to the Bonferroni correction, results with *p* < 0.0042 (i.e., α/m = 0.050/12) were considered statistically significant. ^a^: Within the groups, the differences between week 1 and the follow-ups were statistically significant (*p* < 0.0042), ^b^: Within the groups, the differences between week 2 and the follow-ups were statistically significant (<0.0042), ^c^: Within the groups, the differences between week 3 and the follow-ups were statistically significant (<0.0042).

## Data Availability

The data presented in this study are available on reasonable request from the corresponding author. The data are not publicly available due to privacy and ethical restrictions.
